# Supplementary material for: Red CdSe/ZnS QDs’ Intracellular Trafficking and Its Impact on Yeast Polarization and Actin Filament
Source: Cells. 2023 Feb 2;12(3):484. doi: 10.3390/cells12030484 (PMC9914768; doi:10.3390/cells12030484)
Supplement: Supplementary file 1 [file cells-12-00484-s001.zip › cells-2158496-supplementary.pdf]

Supplementary Data

GFP-Snc1 Distribution Altered by QDs Treatment

Cells expressing GFP-Snc1 were treated with red CdSe/ZnS QDs. The distribution of GFP-Snc1 at the membrane was assessed. The figure below is an extension of Figure 6 in the manuscript with the purpose of providing more visual examples.

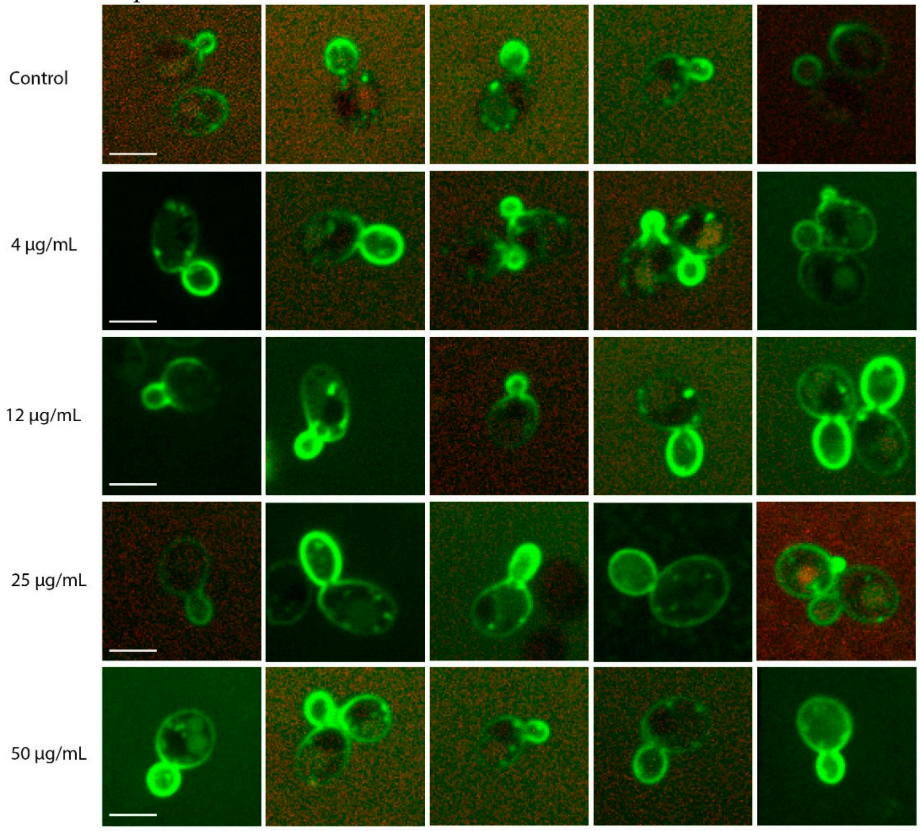

Supplementary Figure S1. The distribution of GFP-Snc1 on the plasma membrane. The size bar is equivalent to 5 µm.
